# Supplementary material for: Virulence Pattern Analysis of Three Listeria monocytogenes Lineage I Epidemic Strains with Distinct Outbreak Histories
Source: Microorganisms. 2021 Aug 16;9(8):1745. doi: 10.3390/microorganisms9081745 (PMC8399138; doi:10.3390/microorganisms9081745)
Supplement: Supplementary file 1 [file microorganisms-09-01745-s001.zip › microorganisms-1311066-supplementary.pdf]

microorganisms

MDPI

Table S1. p-values comparing bacterial loads in the liver of animal infected with the different strains day 3 to 20 post infection (p.i.). $p<0.05$ was considered as statistically significant difference (bold) as determined by the tukey-HSD posthoc test.

Days p.i.	Comparison	PF49	F80594	G6006	EGD
**Day 3**	F80594	0.126	1.000		
	G6006	**0.005**	0.452	1.000	
	EGD	**0.005**	0.448	1.000	1.000
**Day 5**	F80594	0.640	1.000		
	G6006	**0.002**	**0.023**	1.000	
	EGD	0.345	0.953	0.069	1.000
**Day 7**	F80594	**0.000**	1.000		
	G6006	**0.000**	0.261	1.000	
	EGD	**0.002**	0.396	**0.011**	1.000
**Day 10**	F80594	**0.003**	1.000		
	G6006	**0.000**	0.694	1.000	
	EGD	**0.000**	0.694	1.000	1.000
**Day 12**	F80594	0.910	1.000		
	G6006	0.174	0.467	1.000	
	EGD	0.174	0.467	1.000	1.000
**Day 14**	F80594	0.168	1.000		
	G6006	1.000	0.168	1.000	
	EGD	1.000	0.168	1.000	1.000
**Day 20**	F80594	**0.030**	1.000		
	G6006	**0.030**	1.000	1.000	
	EGD	**0.030**	1.000	1.000	1.000
